# Evaluation of C-reactive protein and hematological parameters in smokeless tobacco users: A comparative cross-sectional study

**DOI:** 10.12669/pjms.37.4.3841

**Published:** 2021

**Authors:** Sikander Munir Memon, Naresh Kumar, Aneela Atta Ur Rahman, Binafsha Manzoor Syed

**Affiliations:** 1 Sikander Munir Memon, BDS. Medical Research Centre, Liaquat University of Medical and Health Sciences, Jamshoro 76090, Pakistan; 2 Naresh Kumar, BDS, PhD. Dr. Ishrat Ul Ebad Khan Institute of Oral Health Sciences, Dow University of Health Sciences, Karachi, Pakistan. Medical Research Centre, Liaquat University of Medical and Health Sciences, Jamshoro 76090, Pakistan; 3 Aneela Atta Ur Rahman, MBBS, MPH, PhD. Shaheed Mohtarama Benazir Bhutto Medical University, Larkana 77150, Pakistan. Medical Research Centre, Liaquat University of Medical and Health Sciences, Jamshoro 76090, Pakistan; 4 Binafsha Manzoor Syed, MBBS, PhD. Medical Research Centre, Liaquat University of Medical and Health Sciences, Jamshoro 76090, Pakistan

**Keywords:** C-reactive protein, Cardiovascular disease, Hematological parameters, Smokeless tobacco

## Abstract

**Objective:**

To investigate the changes in levels of C-reactive protein (CRP) and hematological parameters among smokeless tobacco (SLT) users.

**Methods:**

A comparative cross-sectional study was conducted at the community level in the coastal districts of Sindh province namely Badin, Thatta, and Sujawal from January 2017 to December 2019. The CRP and hematological parameters were evaluated by well-established methods among SLT and non-SLT users.

**Results:**

There was a statistically significant difference between SLT users (mean CRP = 0.77) versus non-users (mean CRP = 0.18), p = <0.001. Among hematological parameters, white blood cells (SLT users median = 7.85 versus non-SLT users median = 8.50, p = 0.004), monocytes (SLT users median = 6.00 versus non-SLT users median = 6.00, p = 0.001) and erythrocyte sedimentation rate (SLT users median = 15.00 versus non-SLT users median = 10.00, p = 0.006) showed statistically significant difference.

**Conclusions:**

Significantly elevated CRP was observed in SLT users similarly hematological parameters also showed changes. WBCs, monocytes and ESR were significantly deranged among SLT users. Further studies looking into long term effects of these changes would be helpful.

## INTRODUCTION

Pakistan is a country with one of the highest rate of smokeless tobacco (SLT) users in the world along with many other Asian countries including India and Bangladesh, where people use SLT culturally and traditionally in many forms such as Naswar, Gutka, Paan, Chalia with and without slaked lime and tobacco mix.^1^ Basit et al.2 determined the prevalence of tobacco, ex-tobacco and non-tobacco users in urban/rural areas of four provinces (Punjab, Sindh, Khyber Pakhtunkhwa, and Baluchistan) of Pakistan and they found 13.4% age-standardized prevalence of tobacco use. Extensive use of many precarious chewable tobacco formulations has made oral cancer the second major malignancy in Pakistan.^3^ About 15 million people regularly use tobacco and more than 0.16 million people are killed by diseases caused by tobacco each year. In the current century, tobacco is expected to kill a billion people worldwide.^4^

Globally, more than three quarters of all the SLT consumed by people of South Asian origin; yet there is little evidence on the adverse effects on hematological parameters and levels of C-reactive protein (CRP) caused due to the use of SLT products.^5^ Several researchers have targeted for unique stable and easy to identify biomarkers that can differentiate cancer patients from the healthy and also for the detection of precancerous lesions who are at high risk of developing cancer.^6^

C-Reactive Protein, a standard marker for systemic inflammation is produced in response to inflammatory cytokines in hepatocytes such as TNF-α, IL-1 and IL-6.^7^ The prognostic potency of CRP was validated for patients with colorectal cancer (CRC). Some studies have reported that increased risk of malignancy is associated with the raised levels of CRP and also referred to as factor of prognosis.^8^

Hematological parameters are also reported to be affected widely due to several pharmacological actions of Nicotine and its additives also deteriorate systemic health. Substances present in SLT absorb and act locally on stem cells, keratinocytes and other body tissues. They make adducts of DNA, primarily O-6-methyl-guanine and interfere with the DNA replication and mutation accuracy alternatively add to the molecular chain of events that contribute to malignant cell transformation. Smokeless tobacco substances make the metabolic pattern modular strongly and increase the risk of systemic inflammation, for instance cardiac diseases, polycythemia vera, and modulation of RBC morphology.^9^

Until now, very few studies^10^ have identified the adverse effects on inflammatory markers (acute phase proteins) and blood profiles of the various forms of smokeless tobacco consumption and its proven correlation. A research deficit has been observed with respect to the interaction of SLT with CRP as well as hematological parameters in a population consuming SLT where rationally, SLT is highly prevalent and oral cancer and other systemic diseases are growing at an alarming rate. Therefore, this study was designed with the aim to evaluate CRP and hematological parameters in SLT users.

## METHODS

A comparative cross-sectional study was conducted at the community level in the coastal districts of Sindh province namely Badin, Thatta, and Sujawal from January 2017 to December 2019. The participants were selected using probability multistage sampling technique. Total sample size calculated was 384. The ethical permission was sought from the Research Ethics Committee, Liaquat University of Medical & Health Sciences, Jamshoro, Pakistan before the commencement of this study (Reference: NO. LUMHS/REC/-644, December 26, 2017).

Inclusion criteria for the SLT user group were all individuals aged 15 years and above with a history of more than one year of SLT use (for non-SLT user group, no history of SLT use); no any significant medical condition. Exclusion criteria for the SLT user group as well as the non-SLT user group were, chronic smokers; alcoholics; presence medical disorders.

The questionnaire was filled related to participant’s socio-demographic correlates of SLT. Total 10-ml blood sample per participant was drawn and out of the 10-ml, 2-ml was inserted into EDTA tubes and 7ml blood was inserted into plain tubes and then were sent to LUMHS Diagnostic & Research laboratory for hematological and biochemical analysis under the supervision of consultant pathologist. Specimens were obtained, deposited, transferred, and analyzed according to the approved guideline for body fluid analysis for cellular composition CLSI H56-A.^11^

Data were analyzed using IBM SPSS Statistics for Windows, Version 22.0. Armonk. Frequency and percentage were calculated for the categorical variables. For continuous variables, mean, median and SD were measured. Level of significance was set at p< 0.05.

## RESULTS

A total of 400 participants comprising 200 SLT users and 200 non-SLT users with mean ages of 31.88 and 34.44 years, respectively were analyzed in this study. Demographic characteristics of the SLT users and the non-SLT users are shown in Table-I.

**Table-I:** The demographic characteristics of SLT users and non-SLT users (n=400).

Variable	Frequency (n=400)	Percentage (%)
***SLT Users and Non-SLT users***		
SLT users	200	50.0
Non-SLT users	200	50.0
***Age***		
15 to 20 Years	66	16.5
21 to 30 Years	153	38.3
31 to 40	98	24.5
41 to above	83	20.8
***Gender***		
Male	289	72.3
Female	111	27.8
***Level of Education***		
No schooling	208	52
Primary School	68	17
Secondary School	21	5.3
High School	15	3.8
College	82	20.5
Postgraduate	6	1.5

A Mann-Whitney U test showed that there was a significant difference between the CRP level of the SLT users (mean CRP = 0.77) versus the non-SLT users (mean CRP = 0.18), p <0.001); highlighting the raised levels of CRP in the former (Table-II). Table-III demonstrates variations in the C-reactive protein level in terms of the duration of SLT use.

**Table-II:** The Changes in levels of CRP and Hematological parameters between SLT user and non- SLT user groups. (n=400).

Parameter	SLT users		Non-SLT users		P-value

	(Mean ± SD)	Median (range)	(Mean ± SD)	Median (range)
C-Reactive Protein(mg/dl)	0.77 (1.67)	0.19 (14.41)	0.18 (0.19)	0.10 (0.89)	0.001*
Hemoglobin (gm/dl)	13.91 (1.94)	14.05 (11.90)	13.80 (2.19)	14.10 (13.20)	0.89
Hematocrit (%)	40.60 (5.70)	41.20 (49.30)	40.40 (6.59)	41.65 (40.00)	0.86
Red Blood cells (10^6/L)	5.14 (0.57)	5.15 (3.10)	5.16 (0.65)	5.10 (3.70)	0.88
Mean corpuscular volume FL	78.87 (10.02)	80.75 (67.00)	78.13 (11.15)	80.85 (62.00)	0.90
Mean corpuscular hemoglobin PG	27.41 (5.84)	27.50 (72.30)	26.74 (4.39)	27.80 (23.90)	0.84
Mean corpuscular hemoglobin concentration g/dl	33.64 (1.88)	33.80 (14.20)	34.67 (19.03)	33.30 (277.20)	0.11
White blood cell 10^3/UL	8.99 (7.94)	7.85 (80.70)	9.19 (7.39)	8.50 (105.00)	0.001*
Neutrophils (%)	57.18 (8.91)	58.00 (62.00)	58.00 (8.09)	58.00 (46.00)	0.47
Lymphocytes (%)	31.99 (8.74)	30 (55)	32.03 (7.13)	32 (34)	0.88
Monocytes (%)	5.97 (1.57)	6.00 (8.00)	6.37 (1.43)	6.00 (7.00)	0.001*
Eosinophils (%)	4.00 (2.35)	4 (20)	3.81 (1.64)	4 (19)	0.65
Platelets count x10^9/L	284.76 (77.88)	284.50 (485.00)	288.98 (78.73)	279.00 (543.00)	0.91
Erythrocyte Sedimentation Rate mm/1Hr	15.43 (7.76)	15.00 (55.00)	13.60 (6.86)	10.00 (35.00)	0.001*

* p-value highlights statistically significant difference between SLT and non-SLT user groups.

**Table-III:** Descriptive statistics of C-reactive protein and hematological parameters of the smokeless tobacco users of the study (n=200).

Variable	Duration (in Years)	Min	Max	Mean	Std. Deviation
C-Reactive	1 to 15	0.01	4.39	0.55	0.93
Protein	16 to 30	0.01	14.41	1.04	2.35
	>30	0	5.67	0.68	1.07
Hemoglobin	1 to 15	8.9	18.3	14.22	1.55
	16 to 30	6.4	18.1	13.69	2.26
	>30	8.1	18.3	13.67	1.96
Hematocrit	1 to 15	28.8	48.3	41.83	3.82
	16 to 30	5	52.7	39.60	7.09
	>30	24	54.3	39.90	5.59
Red Blood	1 to 15	3.7	6.8	5.16	0.58
cells	16 to 30	3.8	6.6	5.18	0.59
	>30	4	6	4.97	0.48
Mean	1 to 15	51.2	111	80.54	9.59
corpuscular	16 to 30	44	104.7	76.94	10.84
volume FL	>30	46	97	79.35	8.59
Mean	1 to 15	29.7	37.4	33.79	1.43
corpuscular	16 to 30	24.3	38.5	33.40	2.09
hemoglobin PG	>30	24.3	36.2	33.83	2.30
Mean	1 to 15	15.2	87	28.29	7.52
corpuscular	16 to 30	14.7	40.3	26.52	4.53
hemoglobin	>30	15.5	32.6	27.35	3.18
concentration					
White	1 to 15	3.2	11.7	7.91	1.78
blood cell	16 to 30	2.6	83.3	9.93	11.44
	>30	5.9	50	9.47	7.23
Neutrophils	1 to 15	35	77	58.33	8.44
	16 to 30	18	80	56.79	9.36
	>30	3	70	55.38	8.94
Lymphocytes	1 to 15	0	50	30.67	8.11
	16 to 30	5	52	32.39	8.84
	>30	10	55	34.17	9.75
Monocytes	1 to 15	3	10	6.26	1.51
	16 to 30	2	10	5.9	1.59
	>30	3	9	5.43	1.58
Eosinophils	1 to 15	2	20	4.37	2.67
	16 to 30	0	16	3.78	2.14
	>30	0	10	3.6	1.91
Platelets count	1 to 15	135	590	281.95	84.70
	16 to 30	105	496	273.78	71.46
	>30	160	474	315.57	69.31
Erythrocyte	1 to 15	5	30	15.20	7.18
Sedimentation	16 to 30	5	60	16.5	9.05
Rate	>30	5	25	13.4	5.33

Hematological parameters showed a significant difference for WBCs (SLT-users median= 7.85 versus non-SLT users Median= 8.50, p = 0.004), Monocytes (SLT-users Median= 5.97 versus non-SLT users Median = 6.37, p = 0.001) and ESR (SLT-users Median= 15.00 and non-SLT users Median= 10.00, p = 0.006) (Table-II). In terms of duration-wise, hematological changes due to the use of SLT among three groups has been shown in Table-III.

## DISCUSSION

Smokeless tobacco causes a significant rise in CRP and inflammatory cells. Significant rise of CRP among SLT users can be attributed to the inflammatory response in the body. Our results are in agreement with earlier work, in which authors reported that both smokeless and addictive tobacco use lead to the higher levels of CRP.^12^ In 2000 Furie et al.^13^ also correlated with high levels of CRP with SLT. An increased CRP count is also considered as a blood marker of inflammation, which predicts the risk of myocardial infarction and/or cardiac diseases.

Another important finding from hematological analysis of this study includes a positive relationship between SLT and increased WBC count. Shukla et al.^14^ in their study also reported that the count of leucocytes were higher in SLT users as compared to non-SLT users with the p value of 0.04, which is comparable with the findings of our study. A study performed by Alamgir et al^15^ in Karachi indicated that gene-environment contact is a significant factor, as the chewable types of tobacco are held in the oral pouch for a long time, resulting in increased exposure of the mucosa to carcinogens.

The current study reports a highly significant relationship of SLT with increased counts of monocytes and ESR which indicate the presence of infection in the individuals who consume SLT. Few studies 16,17 confirm the association of SLT with higher levels of monocytes.

SLT is also well known for its effects on physiological processes. SLT user revealed an alteration in RBC morphology. Electron microscopy scanning showed changes in the membranes of RBC with fine “bubble-like” protrusions lacking their discoid shape. SLT ingredients disrupt individuals’ cellular metabolism that contributes to shape changes and scale which have enormous consequences in terms of health maintenance.^14^ In the present study, consumption of SLT has shown an adverse effect on CRP and changes in hematological parameters revealed association with SLT and had systemic adverse effects on the blood profile.

The community-based study with prospective collection of data and central analysis of the markers are strengths of this study. However, the cross-sectional study design without looking into long-term effects of raised hematological markers and CRP are limitations.

The study concludes that elevated levels of CRP are correlated with the use of SLT, and adverse effects on hematological parameters can be seen among SLT users. The findings of this study may help in the early detection of infection, the risk of cardiac disease and the transformation of precancerous lesions into malignancies by conducting more pathways and could serve as an early diagnostic tool in any systemic illness. These findings may also help in designing awareness programs with regard to the deleterious effect of SLT. Further studies with long term follow-up are required to look into effects of raised inflammatory cells and CRP in order to establish systemic effects of Smokeless tobacco.

### Limitations of the study

The assessment was beyond the scope of the analysis of all the toxic components present in different SLT products.

## CONCLUSIONS

The study concludes that elevated levels of CRP are correlated with the use of SLT, and adverse effects on hematological parameters can be seen among SLT users. The findings of this study are likely to help in the early detection of infection, the risk of cardiac disease and the transformation of precancerous lesions into malignancies.

### Authors` Contribution:

**SMM:** Methodology, Data Collection, Data analysis, Original draft preparation, is responsible for integrity of study.

**NK:** Conceptualization, Methodology, Review and editing, Accountable for the accuracy/integrity of the work.

**AAR:** Conceptualization, Methodology, Data analysis, Review and editing.

**BMS:** Data analysis, Review and editing.
